# Supplementary material for: Splice‐dependent trans‐synaptic PTPδ–IL1RAPL1 interaction regulates synapse formation and non‐REM sleep
Source: EMBO J. 2020 Apr 29;39(11):e104150. doi: 10.15252/embj.2019104150 (PMC7265247; doi:10.15252/embj.2019104150)
Supplement: Supplementary file 1 — Appendix [file EMBJ-39-e104150-s001.docx]

*-Appendix-*

**Splice-dependent trans-synaptic PTPδ–IL1RAPL1 interaction regulates synapse formation and NREM sleep**

Haram Park^1,7^, Yeonsoo Choi^1,7^, Hwajin Jung^1,7^, Seoyeong Kim^2,7^, Suho Lee^1^, Hyemin Han^3^, Hanseul Kweon^2^, Suwon Kang^2^, Woong Seob Sim^2^, Frank Koopmans^4,5^, Esther Yang^6^, Hyun Kim^6^, August B. Smit^5^, Yong Chul Bae^3^, Eunjoon Kim^1,2,#^

^1^Center for Synaptic Brain Dysfunctions, Institute for Basic Science (IBS), Daejeon 34141, Korea; ^2^Department of Biological Sciences, Korea Advanced Institute for Science and Technology (KAIST), Daejeon 34141, Korea; ^3^Department of Anatomy and Neurobiology, School of Dentistry, Kyungpook National University, Daegu 700-412, Korea; ^4^Department of Functional Genomics, CNCR, VU University and UMC Amsterdam, 1081 HV Amsterdam, the Netherlands; ^5^Department of Molecular and Cellular Neurobiology, CNCR, VU University and UMC Amsterdam, 1081 HV Amsterdam, the Netherlands; ^6^Department of Anatomy and Division of Brain Korea 21, Biomedical Science, College of Medicine, Korea University, Seoul 136-705, Korea; ^7^These authors contributed equally to the work; ^#^Corresponding author: kime@kaist.ac.kr

**Table of Contents**

**Appendix Figure S1**

**Appendix Figure S1 Legend**

**p1**

**p2**

**
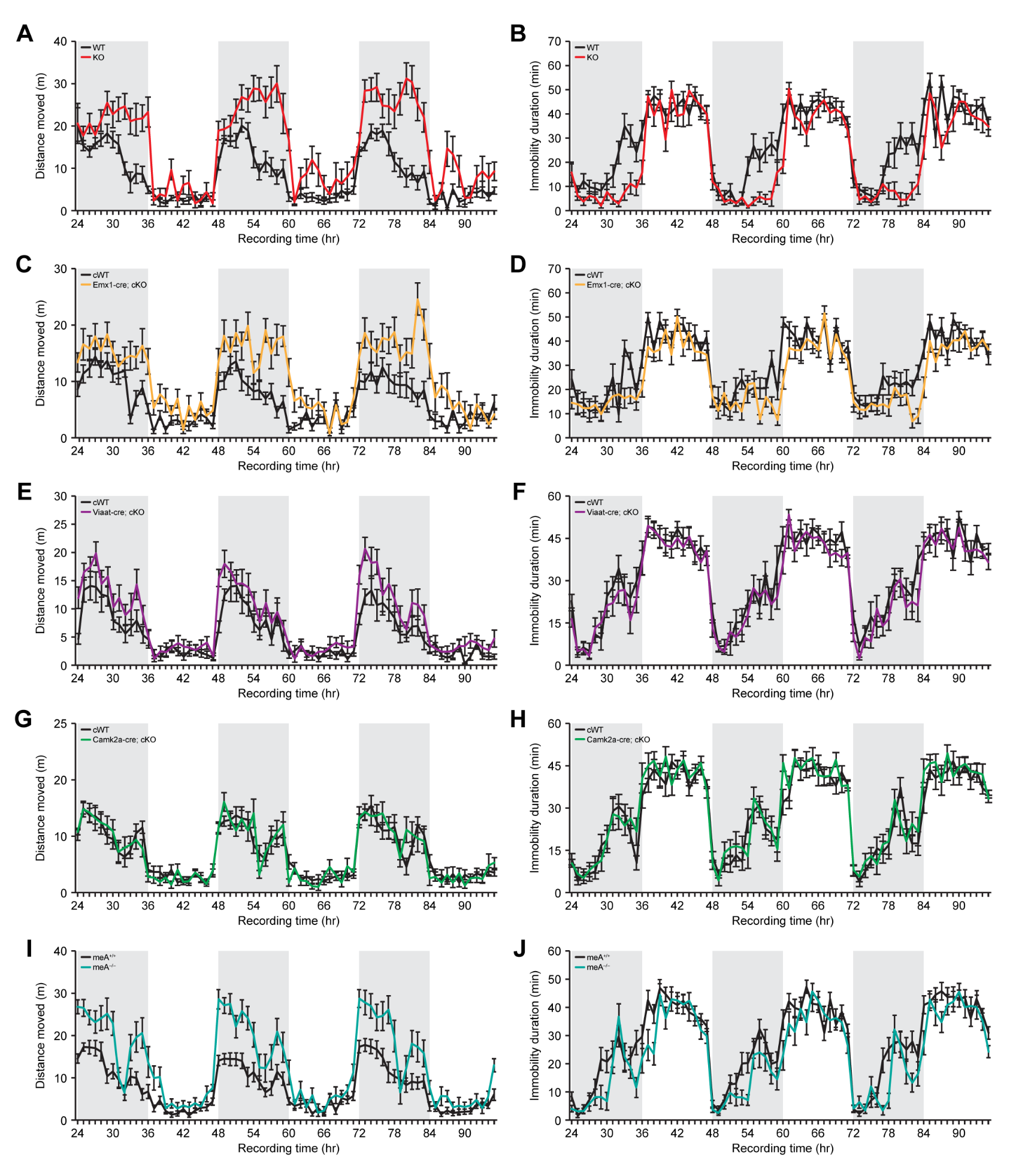
**

## Appendix Figure S1. Complete hourly Laboras data for the shared hyperactivity and sleep-dysfunction phenotypes of Ptprd–/–, Emx1-cKO, and Ptprd-meA–/– mice.

(A–J) Hyperactivity of *Ptprd^–/–^* mice (2.5–4 months) in Laboras cages observed during the light-off periods and light-on periods, mimicked strongly by *Emx1*-cKO mice, but weakly by *Viaat*-cKO mice and not at all by *Camk2a*-cKO mice. (n = 14 mice [WT], 14 [global KO], 16 [*Emx1*-cWT], 16 [*Emx1*-cKO], 11 [*Viaat*-cWT], 13 [*Viaat*-cKO], 18 [*Camk2a-*cWT], 13 [*Camk2a-*cKO], 16 [*Ptprd-meA^+/+^*], and 15 [*Ptprd-meA^–/–^*]).
