# Supplementary material for: Splice‐dependent trans‐synaptic PTPδ–IL1RAPL1 interaction regulates synapse formation and non‐REM sleep
Source: EMBO J. 2020 Apr 29;39(11):e104150. doi: 10.15252/embj.2019104150 (PMC7265247; doi:10.15252/embj.2019104150)
Supplement: Supplementary file 2 — Expanded View Figures PDF [file EMBJ-39-e104150-s002.pdf]

Expanded View Figures

Figure EV1. Distribution patterns of PTPδ-tdTomato protein in brain regions and at subcellular and ultrastructural sites in PTPδ-tdTomato reporter mice.

A–C Distribution patterns of PTPδ-tdTomato protein revealed by confocal microscopic imaging of coronal, horizontal, and sagittal sections of brains from PTPδ-tdTomato reporter mice (P21). The imaging used the PTPδ-tdTomato signals from unstained brain slices. Red tdTomato signals were converted to grayscale for enhanced visibility. Dotted boxes indicate location of corresponding panels of Fig 1C. Scale bar, 2 mm.

D PTPδ-tdTomato signals are mainly detected in axonal compartments of mature (DIV 24) cultured hippocampal and cortical (entorhinal cortex; 2:1 mixture) glutamatergic and GABAergic neurons, marked by vGluT1 and GAD67, respectively. Scale bar, 10 μm in main images and 2 μm in enlarged images.

E PTPδ-tdTomato signals are detected in both tau-positive axons and MAP2-positive dendrites in immature (DIV 6) cultured hippocampal and cortical (entorhinal cortex; 2:1 mixture) neurons. Scale bar, 50 μm in main images and 10 μm in enlarged images.

F Quantification of the results in Fig 1E for colocalization of PTPδ-tdTomato with PSD-95 and gephyrin clusters. For quantification, the area, average intensity, or total intensity of tdTomato signals in areas of tdTomato clusters overlapping with the areas of PSD-95/gephyrin clusters was normalized to those (area/average intensity/total intensity) of total tdTomato clusters to obtain the PTPδ area/intensity/total ratio (*y*-axis) of WT and KI at excitatory/inhibitory synapses (n = 15 images [WT and KO], mean ± SEM, ***$P < 0.001$, ns, not significant, Mann-Whitney U test).

G–I Electron micrographs showing the ultrastructural distribution patterns of PTPδ-tdTomato DAB signals in the SLM region of the hippocampus (P21) juxtaposed to PSD structures and colocalized with vGlut2 immunogold particles (excitatory axon terminals) but not with GAD67/65 immunogold particles (inhibitory axon terminals). Inset regions (red squares) are enlarged in main Fig 1F–H. Scale bar, 500 nm.

J PTPδ and PTPδ-tdTomato proteins similarly distribute to subcellular brain fractions, as shown by immunoblot analysis of subcellular fractions from the whole brain of PTPδ-tdTomato reporter mice (P21). Analysis of immunoblots reveals no difference in the subcellular localization of PTPδ-tdTomato fusion proteins as compared to wild-type PTPδ. H, Total homogenate; P1, nuclei and large debris; S2, supernatant after P2 precipitation; P2, crude synaptosomes; S3, cytosol; P3, small membrane; LP1, synaptosomal membrane; LS2, synaptic cytosol; LP2, synaptic vesicles (n = 3 mice for each fraction, mean ± SEM, ns, not significant, two-way RM ANOVA with Holm-Sidak test).

Source data are available online for this figure.

 © 2020 The Authors

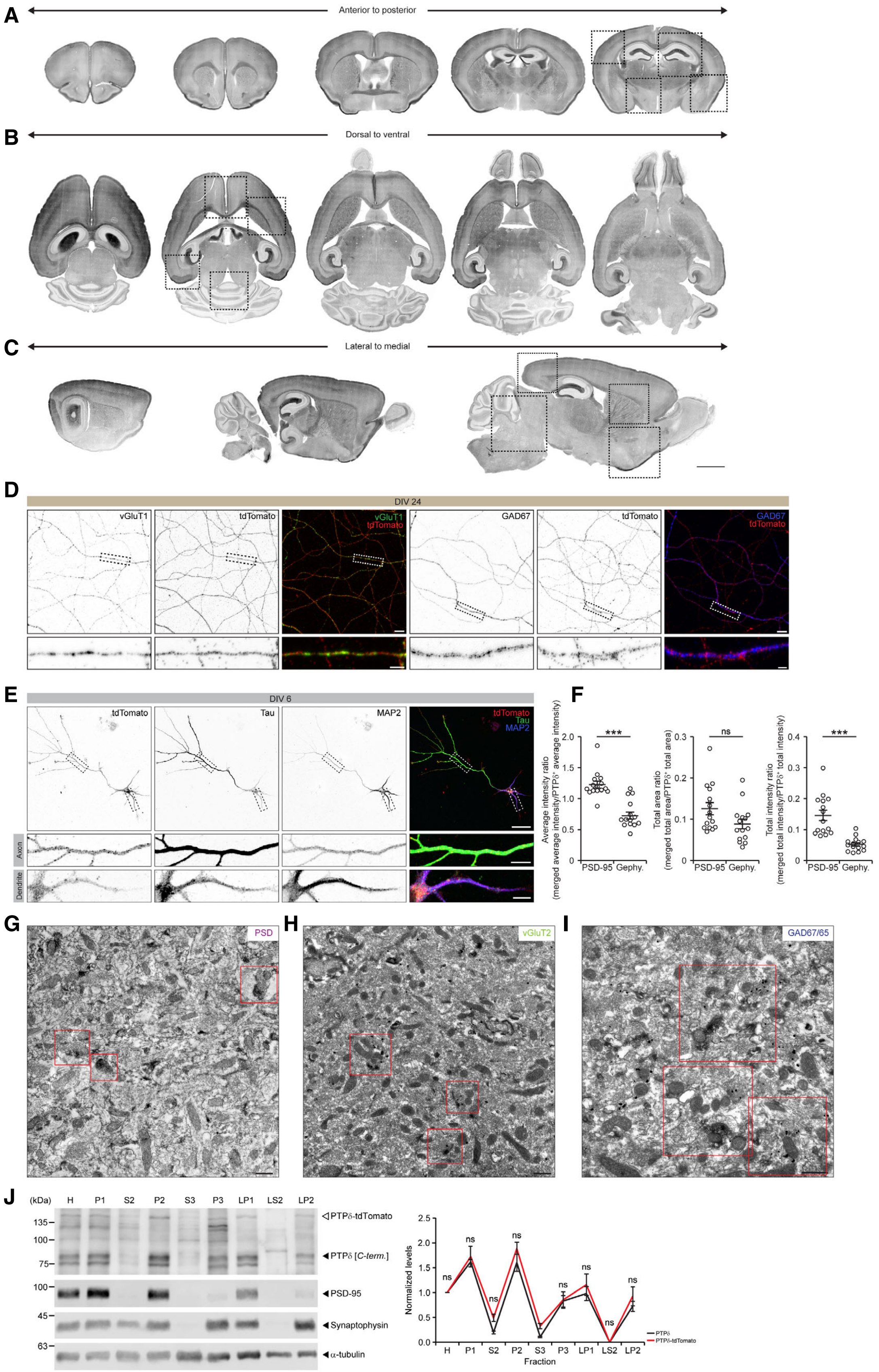
A
Anterior to posterior
B
Dorsal to ventral
C
Lateral to medial
D
DIV 24
vGluT1
tdTomato
GAD67
E
DIV 6
Tau
MAP2
Axon
Dendrite
F
Average intensity ratio (merged average intensity/PTPδ+ average intensity)
Total area ratio (merged total area/PTPδ+ total area)
Total intensity ratio (merged total intensity/PTPδ+ total intensity)
PSD-95
Gephy.
***
ns
G
PSD
H
vGluT2
I
GAD67/65
J
(kDa)
H P1 S2 P2 S3 P3 LP1 LS2 LP2
PTPδ-tdTomato
PTPδ [C-term.]
PSD-95
Synaptophysin
α-tubulin
Normalized levels
Fraction
PTPδ
PTPδ-tdTomato

Figure EV1.

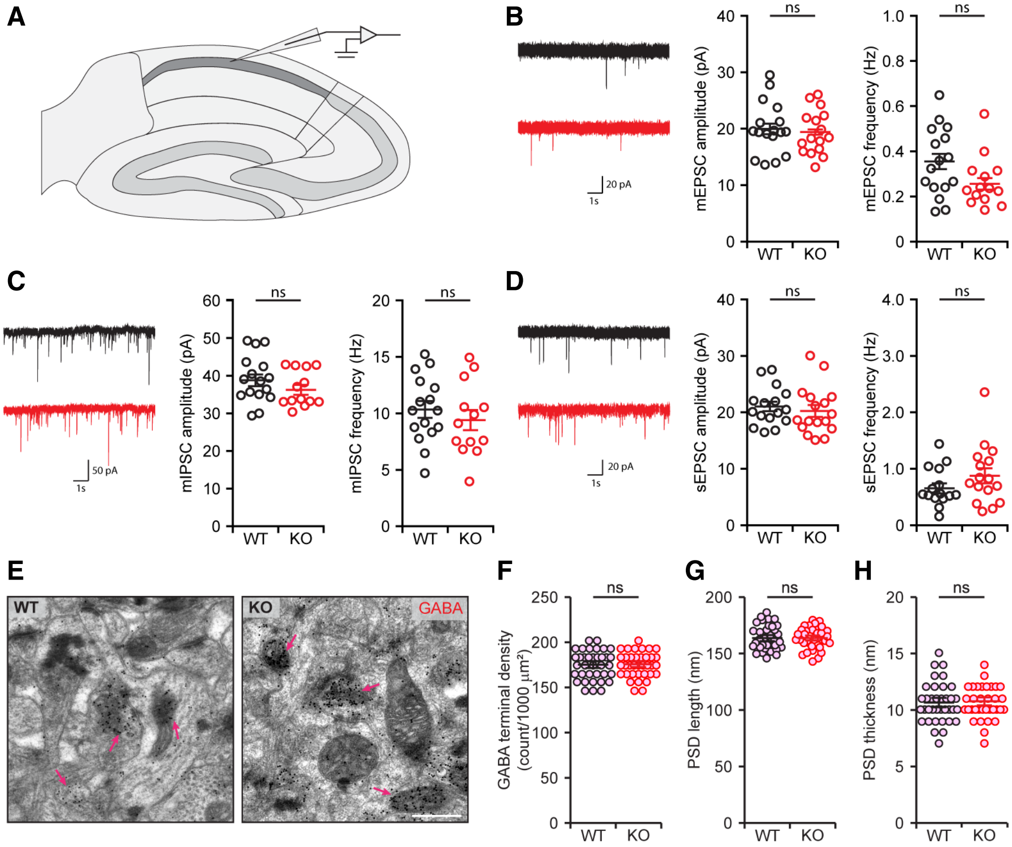

Figure EV2. Normal levels of spontaneous synaptic transmission in *Ptprd*$^{-/-}$ CA1 neurons and GABAergic synapse density and structure in the *Ptprd*$^{-/-}$ SLM.

A Schematic diagrams for the recording of spontaneous synaptic transmission in CA1 pyramidal neurons.

B Normal mEPSC frequency and amplitude in *Ptprd*$^{-/-}$ CA1 pyramidal neurons (P21–24). Analysis of frequency and amplitude of mEPSC peaks reveal no statistical difference between WT and *Ptprd*$^{-/-}$ mice (n = 17 neurons from 3 mice [WT], 16, 3 [KO], mean ± SEM, ns, not significant, Student's t-test).

C Normal mIPSCs in CA1 pyramidal neurons of *Ptprd*$^{-/-}$ mice (P22–24). Analysis of frequency and amplitude of mIPSC peaks reveal no statistical difference between WT and *Ptprd*$^{-/-}$ mice (n = 16 neurons from 3 mice [WT], 13, 3 [KO], mean ± SEM, ns, not significant, Student's t-test).

D Normal sEPSCs in CA1 pyramidal neurons of *Ptprd*$^{-/-}$ mice (P21–25). Analysis of frequency and amplitude of sEPSC peaks reveal no statistical difference between WT and *Ptprd*$^{-/-}$ mice (n = 16 neurons from 4 mice [WT], 17, 4 [KO], mean ± SEM, ns, not significant, Mann-Whitney U test).

E–H Normal density, length, and thickness of GABAergic synapses in the *Ptprd*$^{-/-}$ SLM (P21), as revealed by EM measurement of PSDs (pink arrowheads) apposed to GABA-positive axon terminals, marked by immunogold staining for GABA (red arrows) (n = 36 images from 3 mice [WT], 36, 3 [KO], mean ± SEM, ns, not significant, Student's t-test, Mann-Whitney U test). Scale bar, 500 nm.

 © 2020 The Authors

Figure EV3. Largely normal total levels of synaptic proteins in hippocampal *Ptprd*$^{-/-}$ SLM and SR regions and unaltered synaptic enrichment of Slitrk3, SALM3, and NGL-3 in the *Ptprd*$^{-/-}$ brain.

A Schematic diagram for the dissection of SR and SLM layers in the hippocampus (P21–27).

B–F Representative immunoblots of total lysates from the SR and SLM layers for the tested synaptic proteins, including proteins known to be enriched in the SLM (NGL-1 and HCN1), the PTPδ relative PTPσ, postsynaptic partners of PTPδ (IL1RAPL1, Slitrk2/3, and NGL-3), presynaptic scaffolds/adaptors (Bassoon and liprin-α), postsynaptic scaffolds/adaptors (CaMKIIα/β, PSD-95, SynGAP1), postsynaptic receptors (GluA1/2, GluN1, GluN2A/B), and signaling molecules (phospho-Src). α-Tubulin was used as a control (n = 4 mice for WT and KO, mean ± SEM, ***$P < 0.001$, ns, not significant, two-way ANOVA, Tukey's HSD post-hoc test).

G Normal synaptic levels of other PTPδ-binding partners (Slitrk3, SALM3, and NGL-3) in the *Ptprd*$^{-/-}$ brain (P21–27), as revealed by immunoblotting of crude synaptosomal (P2), synaptic plasma membrane (SPM), and PSD (PSD II) fractions (n = 3 mice [WT and KO] for each fraction [P2, SPM, and PSD], mean ± SEM, ns, not significant, Student's t-test).

H Normal synaptic levels of other PTPδ-binding partners (Slitrk3, SALM3, and NGL-3) in the *Ptprd-meA*$^{-/-}$ brain (P21–27), as revealed by immunoblotting of P2, SPM, and PSD (PSD II) fractions (n = 3 mice [*Ptprd-meA*$^{+/+}$ and *Ptprd-meA*$^{-/-}$] for each fraction [P2, SPM, and PSD], mean ± SEM, ns, not significant, Student's t-test).

Source data are available online for this figure.

© 2020 The Authors

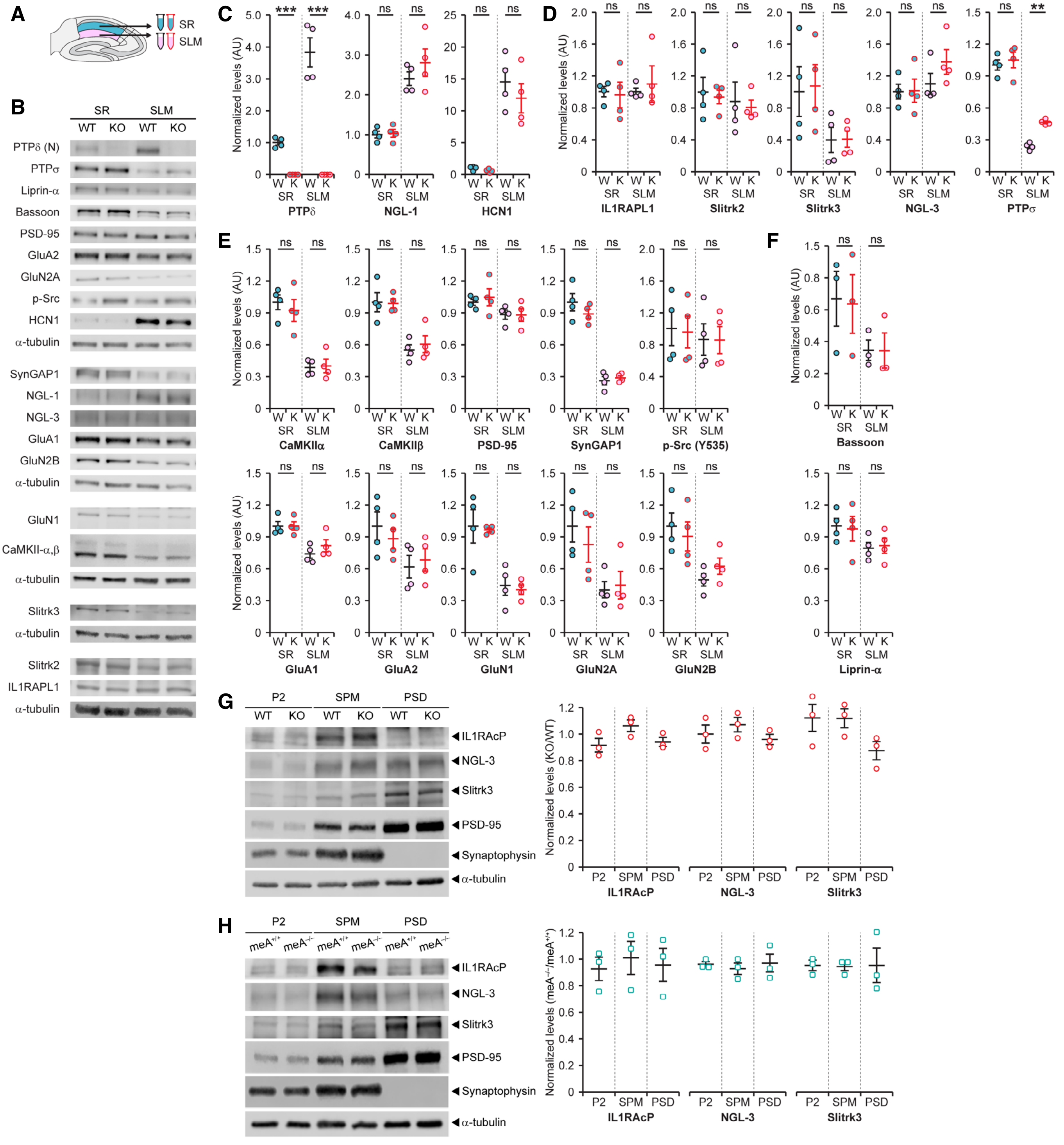

Figure EV3.

 © 2020 The Authors

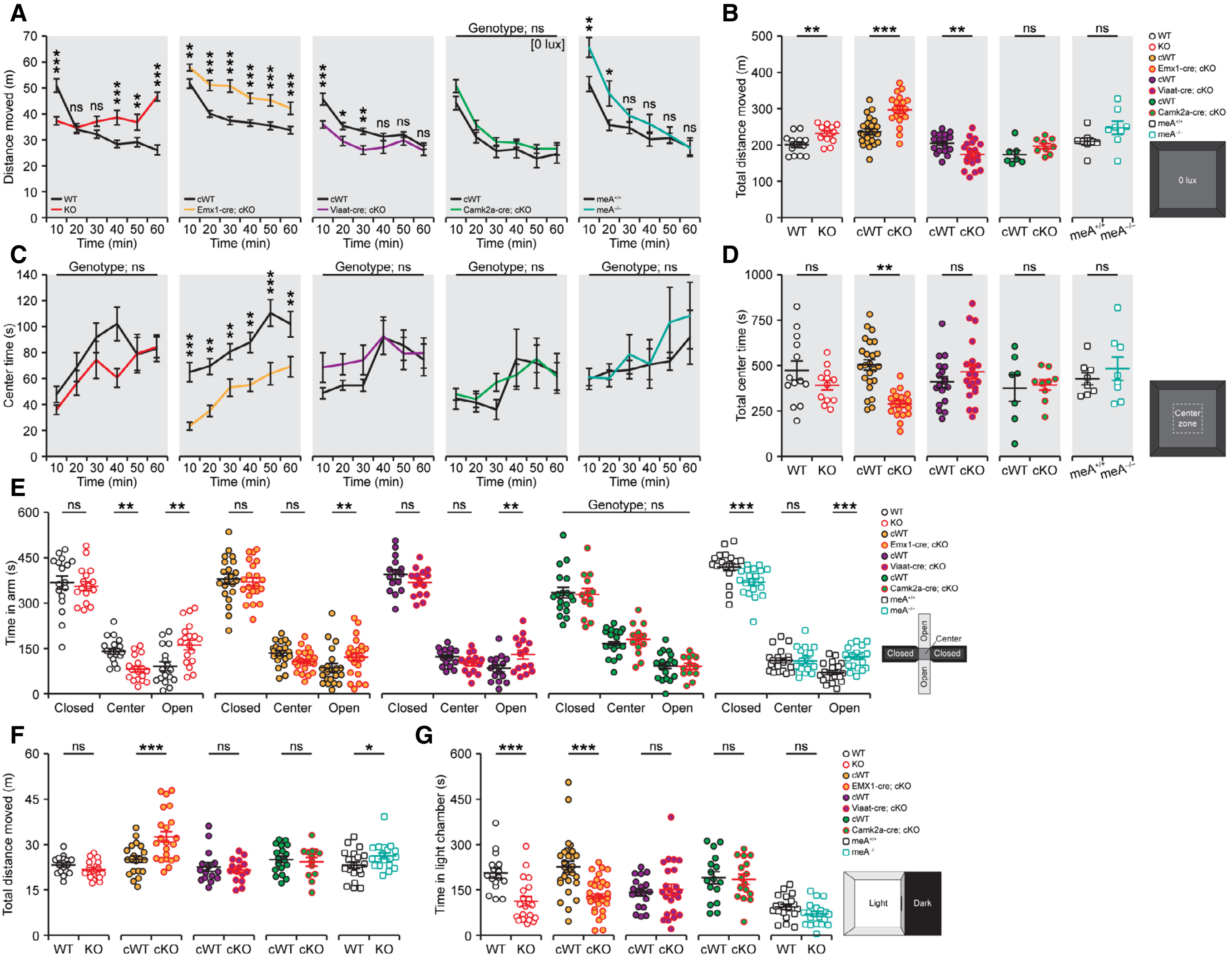

Figure EV4. Hyperactivity and anxiety-like behaviors of *Ptprd*$^{-/-}$, *Emx1*-cKO, *Viaat*-cKO, *CaMKII*-cKO, and *Ptprd-meA*$^{-/-}$ mice in open-field, elevated plus-maze, and light–dark tests.

A–D Hyperactivity of global *Ptprd*$^{-/-}$, *Emx1*-cKO, *Viaat*-cKO, *Camk2a*-cKO, and *Ptprd-meA*$^{-/-}$ mice (2.5–4 months) in the open-field test, indicated by distance moved and total distance moved. Note that the hyperactivity of global *Ptprd*$^{-/-}$ mice is mimicked by *Emx1*-cKO mice, but not by *Viaat*-cKO, *Camk2a*-cKO, or *Ptprd-meA*$^{-/-}$ mice. Note also that *Emx1*-cKO, but not other mice, showed enhanced anxiety-like behaviors, as indicated by the time spent in the center region of the open-field arena (n = 13 mice [WT], 12 [global KO], 24 [*Emx1*-cWT], 19 [*Emx1*-cKO], 18 [*Viaat*-cWT], 20 [*Viaat*-cKO], 7 [*Camk2a*-cWT] and 9 [*Camk2a*-cKO], 8 [*Ptprd-meA*$^{+/+}$] and 8 [*Ptprd-meA*$^{-/-}$], mean ± SEM, *P < 0.05, **P < 0.01, ***P < 0.001, ns, not significant, two-way RM ANOVA with Holm-Sidak test for A and B, Student's t-test for all pairs in B and D expect for Mann-Whiney U test for the *Emx1* pair).

E, F Anxiety-like behaviors in *Ptprd*$^{-/-}$, *Emx1*-cKO, *Viaat*-cKO, *Camk2a*-cKO, and *Ptprd-meA*$^{-/-}$ mice (2.5–4 months) in the elevated plus-maze test, as indicated by time spent in closed/open arms and the center. Note that *Ptprd*$^{-/-}$, *Emx1*-cKO, and *Viaat*-cKO mice, but not *Camk2a*-cKO or *Ptprd-meA*$^{-/-}$, mice show slight anxiolytic-like behavior. Note also that *Emx1*-cKO and *Ptprd-meA*$^{-/-}$ mice show hyperactivity in the EPM (F) (n = 13 mice [WT], 12 [global KO], 24 [*Emx1*-cWT], 19 [*Emx1*-cKO], 18 [*Viaat*-cWT], 20 [*Viaat*-cKO], 7 [*Camk2a*-cWT] and 9 [*Camk2a*-cKO], 20 [*Ptprd-meA*$^{+/+}$], and 19 [*Ptprd-meA*$^{-/-}$], mean ± SEM, *P < 0.05, **P < 0.01, ***P < 0.001, ns, not significant, two-way RM ANOVA with Holm-Sidak test for E, Student's t-test for all pairs in F except for Mann-Whitney U test for the *Emx1* pair).

G Anxiety-like behaviors in *Ptprd*$^{-/-}$, *Emx1*-cKO, *Viaat*-cKO, *Camk2a*-cKO, and *Ptprd-meA*$^{-/-}$ mice (2.5–4 months) in the light–dark test, as indicated by time spent in light chamber. Note that *Ptprd*$^{-/-}$ and *Emx1*-cKO mice, but not *Viaat*-cKO, Camk2a-cKO, or *Ptprd-meA*$^{-/-}$ mice, show enhanced anxiety-like behavior (n = 16 mice [WT], 20 [global KO], 28 [*Emx1*-cWT], 31 [*Emx1*-cKO], 18 [*Viaat*-cWT], 23 [*Viaat*-cKO], 16 [*Camk2a*-cWT] and 16 [*Camk2a*-cKO], 20 [*Ptprd-meA*$^{+/+}$], and 20 [*Ptprd-meA*$^{-/-}$], mean ± SEM, ***P < 0.001, ns, not significant, Student's t-test for *Viaat*, *Camk2a*, and *Ptprd-meA* pairs, Mann-Whitney U test for *Ptprd*$^{-/-}$ and *Emx1* pairs).

© 2020 The Authors

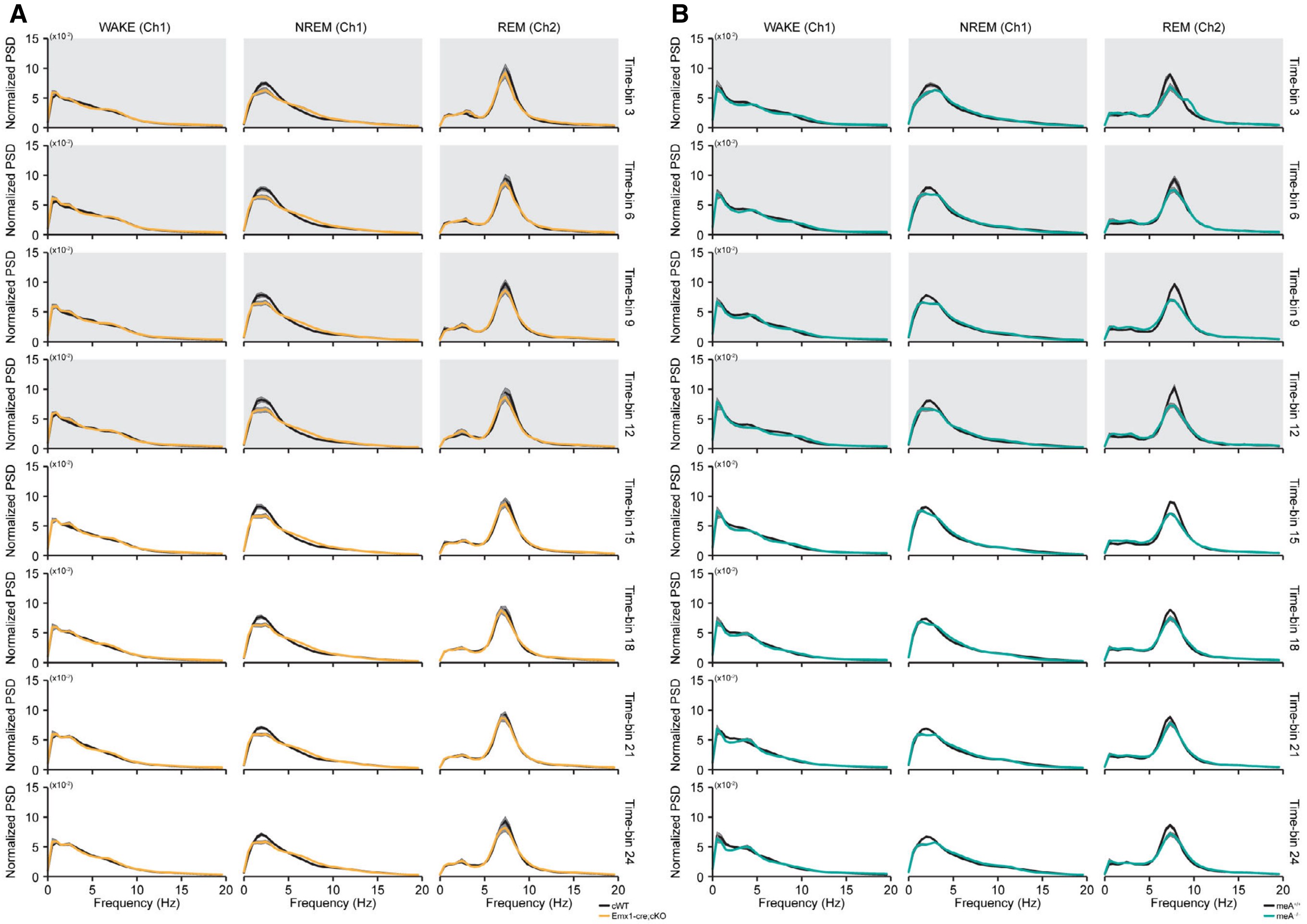

Figure EV5. Raw EEG data from *Emx1-Cre;Ptprd*$^{fl/fl}$ and *Ptprd-meA*$^{-/-}$ mice.

A, B Normalized power spectrogram (0–20 Hz) of each time-bin (rows) and brain states in *Emx1*-cKO mice (A) and *Ptprd-meA*$^{-/-}$ mice (B) (2.5–4 months). Gray backgrounds in A and B panels denote light-off hours (n = 9 mice [*Emx1*-cWT], 9 [*Emx1*-cKO], 8 [*Ptprd-meA*$^{+/+}$], and 8 [*Ptprd-meA*$^{-/-}$]).

 © 2020 The Authors
