# Supplementary figures and images for: Splice‐dependent trans‐synaptic PTPδ–IL1RAPL1 interaction regulates synapse formation and non‐REM sleep
Source: EMBO J. 2020 Apr 29;39(11):e104150. doi: 10.15252/embj.2019104150 (PMC7265247; doi:10.15252/embj.2019104150)

FigEV1.

FigEV1. J

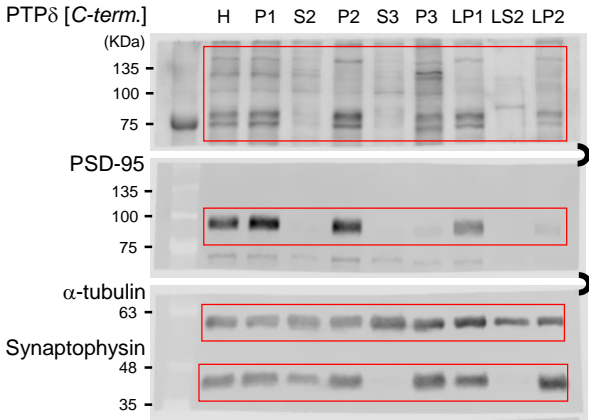

Supplement: Supplementary file 5 — Source Data for Expanded View [file EMBJ-39-e104150-s009.zip › EV_Figure_Source_Data/Figure_EV1_Source_Data.pdf]

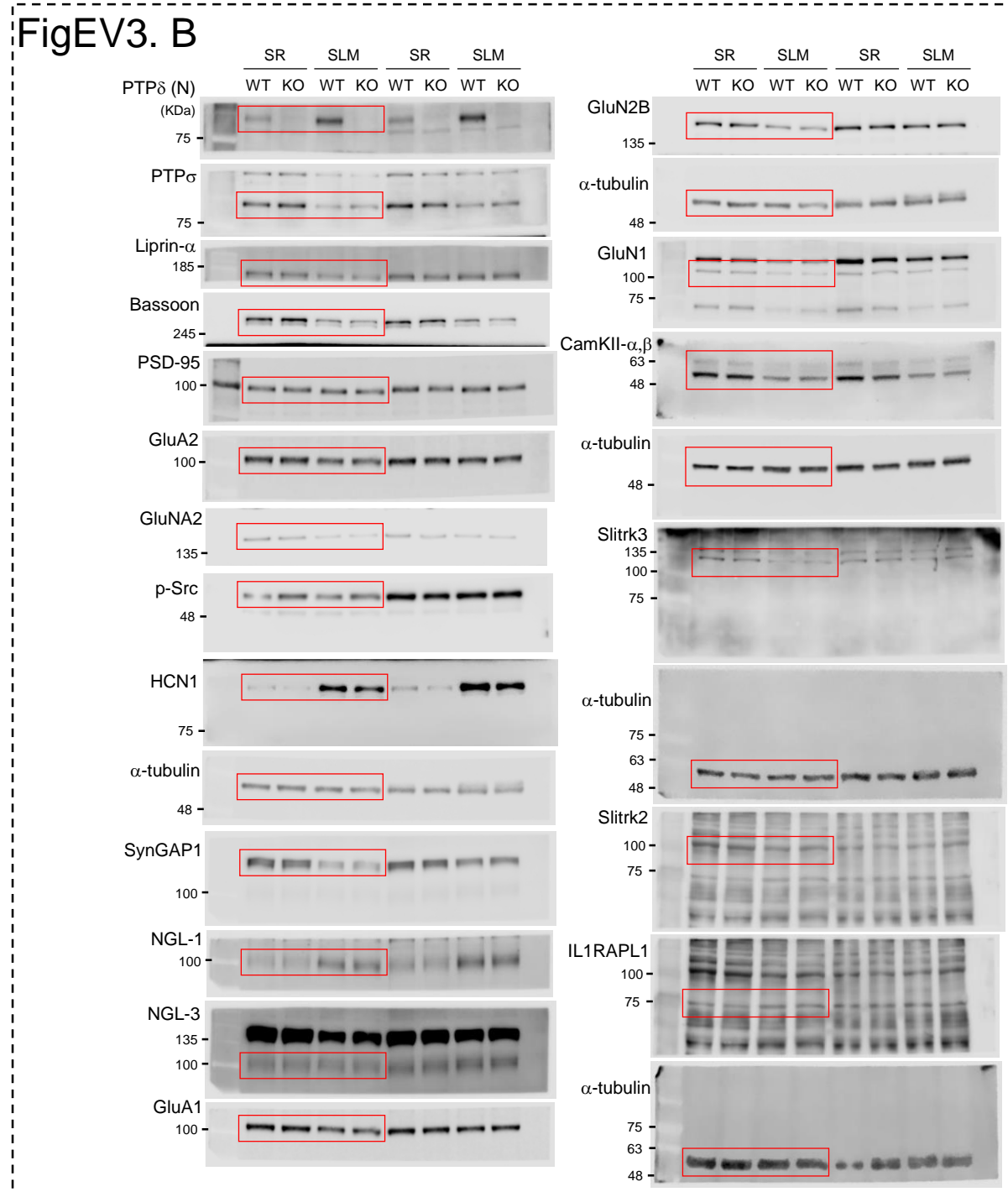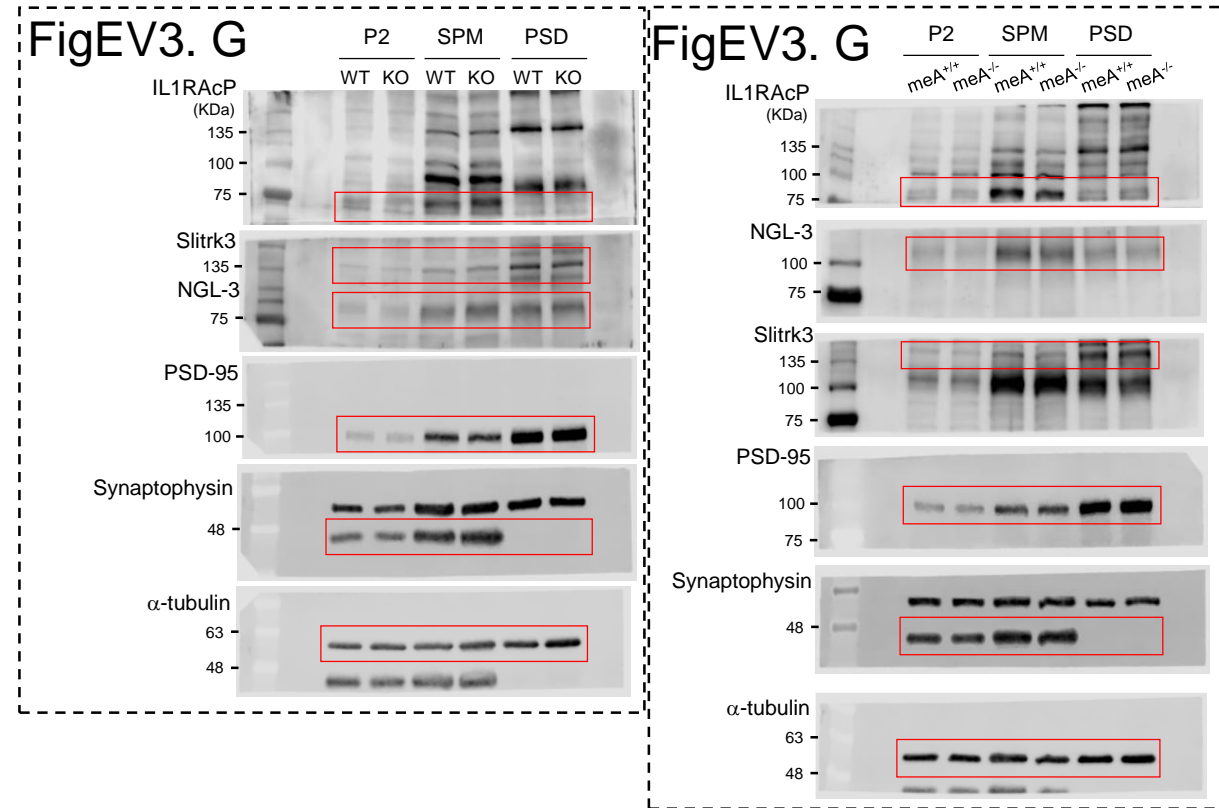

Supplement: Supplementary file 5 — Source Data for Expanded View [file EMBJ-39-e104150-s009.zip › EV_Figure_Source_Data/Figure_EV3_Source_Data.pdf]

Fig1.

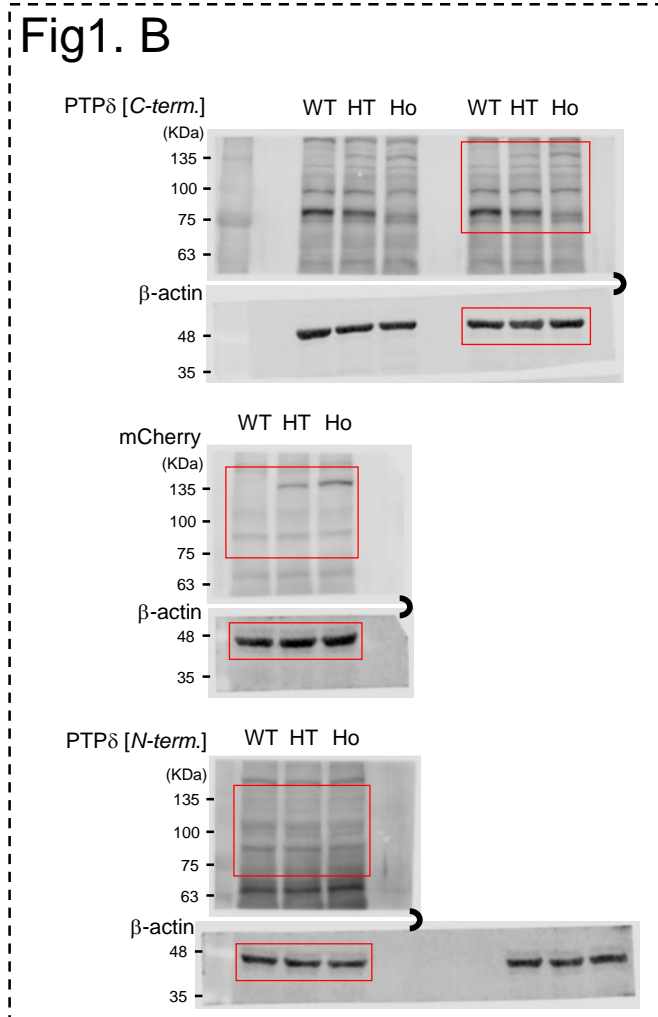

Supplement: Supplementary file 7 — Source Data for Figure 1 [file EMBJ-39-e104150-s005.pdf]

Fig2.

Fig2. B

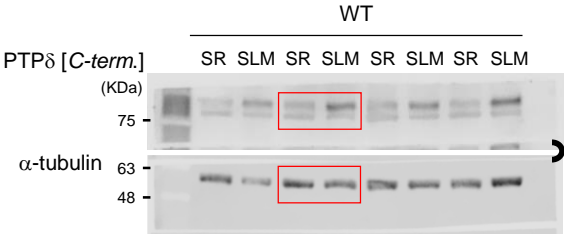

Fig2. D

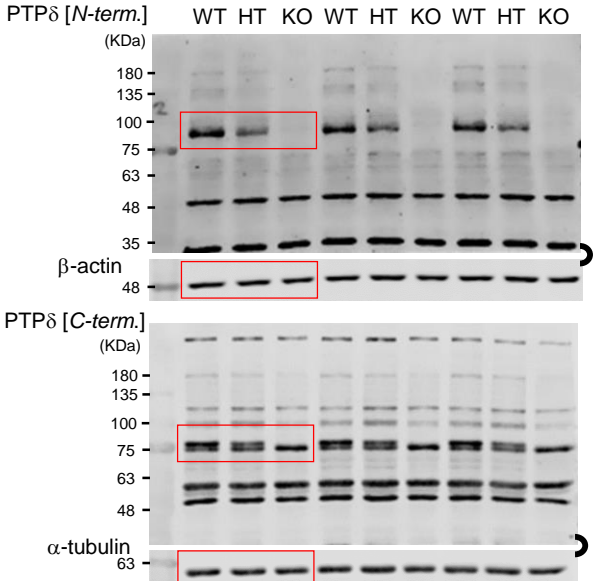

Supplement: Supplementary file 8 — Source Data for Figure 2 [file EMBJ-39-e104150-s006.pdf]

Fig4.

Fig4.A

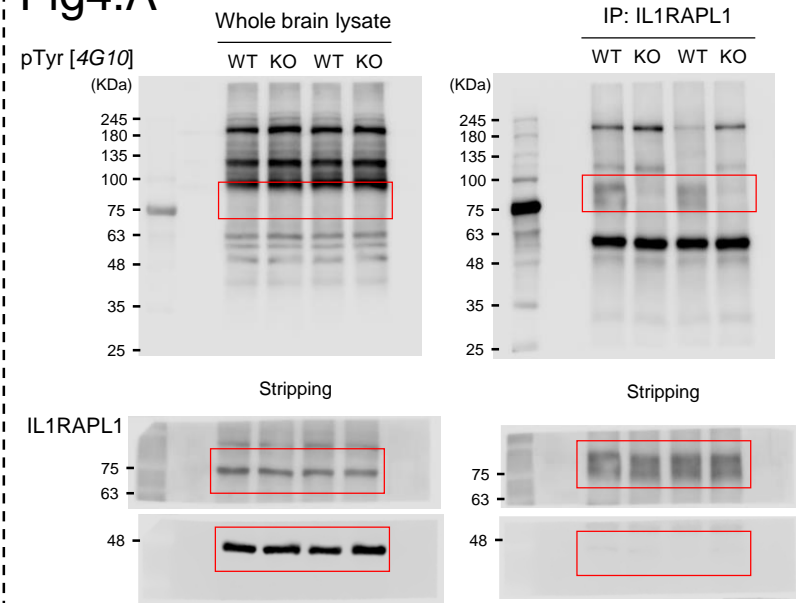

Fig4.B

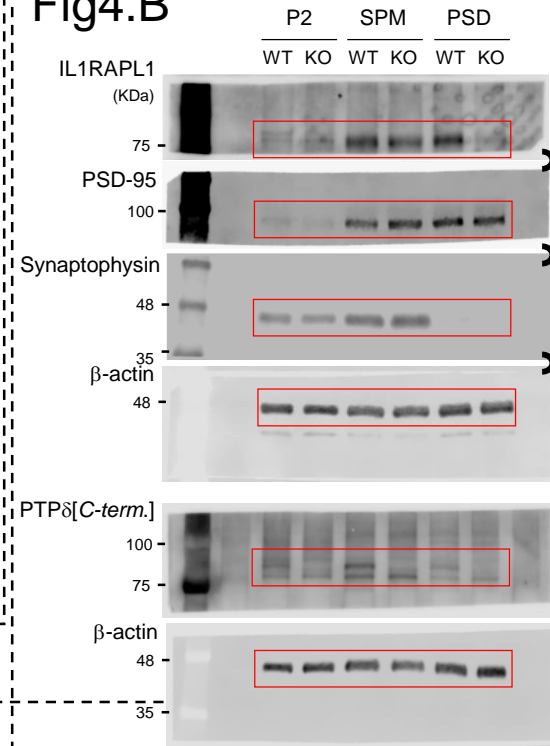

Fig4.F

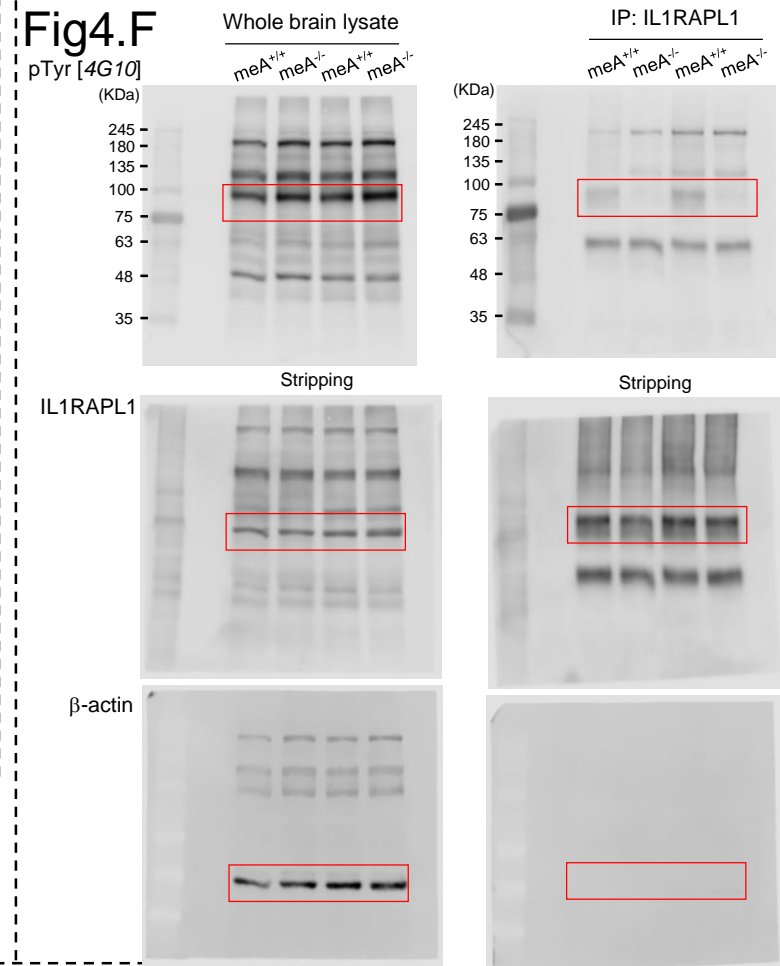

Fig4.G

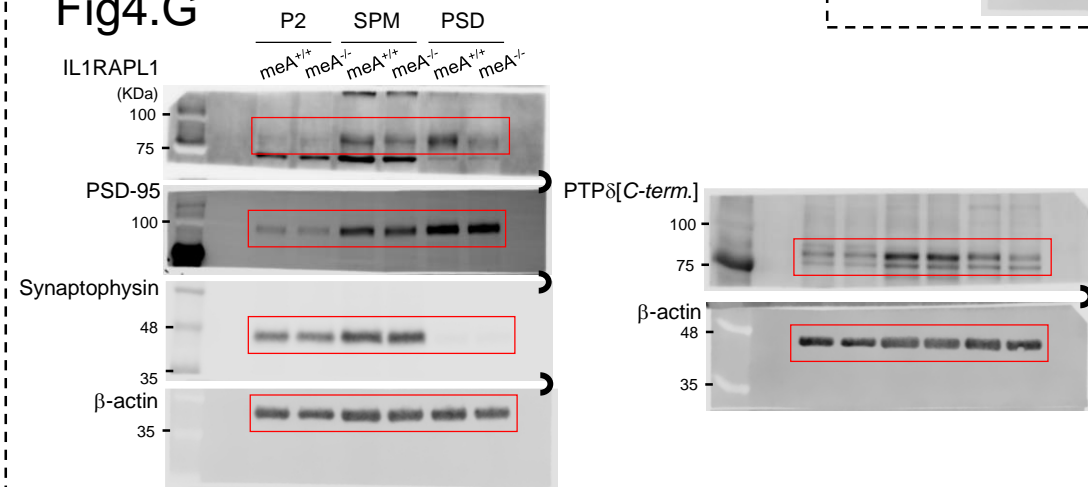

Fig4.E

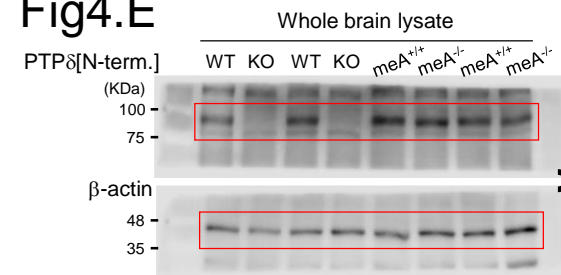

Supplement: Supplementary file 10 — Source Data for Figure 4 [file EMBJ-39-e104150-s008.pdf]
